# Small molecule perturbation of the CAND1-Cullin1-ubiquitin cycle stabilizes p53 and triggers Epstein-Barr virus reactivation

**DOI:** 10.1371/journal.ppat.1006517

**Published:** 2017-07-17

**Authors:** Nadezhda Tikhmyanova, Steve Tutton, Kayla A. Martin, Fang Lu, Andrew V. Kossenkov, Nicholas Paparoidamis, Shannon Kenney, Joseph M. Salvino, Paul M. Lieberman

**Affiliations:** 1 The Wistar Institute, Program in Gene Expression and Regulation, Philadelphia, Pennsylvania, United States of America; 2 Drexel University, College of Medicine, Department of Pharmacology, Philadelphia, Pennsylvania, United States of America; 3 The University of Wisconsin, School of Medicine and Public Health at Madison, McCardle Laboratory for Cancer Research, Departments of Oncology and Medicine, Madison, Wisconsin, United States of America; Tulane Health Sciences Center, UNITED STATES

## Abstract

The chemical probe C60 efficiently triggers Epstein-Barr Virus (EBV) reactivation from latency through an unknown mechanism. Here, we identify the Cullin exchange factor CAND1 as a biochemical target of C60. We also identified CAND1 in an shRNA library screen for EBV lytic reactivation. Gene expression profiling revealed that C60 activates the p53 pathway and protein analysis revealed a strong stabilization and S15 phosphorylation of p53. C60 reduced Cullin1 association with CAND1 and led to a global accumulation of ubiquitylated substrates. C60 also stabilized the EBV immediate early protein ZTA through a Cullin-CAND1-interaction motif in the ZTA transcription activation domain. We propose that C60 perturbs the normal interaction and function of CAND1 with Cullins to promote the stabilization of substrates like ZTA and p53, leading to EBV reactivation from latency. Understanding the mechanism of action of C60 may provide new approaches for treatment of EBV associated tumors, as well as new tools to stabilize p53.

## Introduction

Epstein-Barr Virus (EBV) is a human gammaherpesvirus that establishes latent infection in B-lymphocytes in over 90% of adults worldwide [1]. EBV latent infection is also associated with ~1% of all human cancers, including various forms of Burkitt lymphoma (BL), nasopharyngeal carcinoma, Hodgkin’s and non-Hodgkin’s lymphoma, NK/T cell lymphoma and gastric carcinoma [2–4]. Antiviral agents targeting DNA replication enzymes of human herpesviruses are effective at inhibiting productive infection, but to date there are no approved therapeutics for treatment of latent infection and its associated malignancies [3]. An alternative strategy has been to induce lytic cycle gene expression and replication, to be followed by treatment with antivirals, such as ganciclovir, that kill lytic infected cells [5–8]. Lytic inducers have also been used to enhance the efficacy of immune therapies, such as therapeutic vaccines and adoptive T-cells [9].

EBV can be reactivated through multiple pathways and cellular stress responses [10–12]. In lymphocytes, transcription activation of the immediate early gene BZLF1, encoding the bZIP transcriptional activator ZTA (also referred to as ZEBRA and Z), is sufficient to trigger the viral lytic cycle [11, 13]. BZLF1 transcription can be activated partially by phorbol esters through PKC, ERK and MAP kinase pathways, calcium ionophores through calcineurin and NFAT pathways, and HDAC inhibitors through reversal of epigenetic silencing at the BZLF1 promoter [10–12, 14]. The DNA-damage response pathway involving ATM and p53 activation has also been implicated in the reactivation of EBV [12, 15, 16]. While the pathways that activate the BZLF1 promoter have been investigated extensively, relatively less is known about the mechanisms that regulate ZTA protein function and stability, and whether this can also be modulated to control the reactivation process.

The ZTA transcriptional activation domain is subject to several modifications and interactions that may modulate its function and stability. ZTA can be SUMOylated on lysine 12 to down-regulate its transcription activation function, and potentiate its DNA replication function, through mechanisms not completely understood [17–19]. The ZTA activation domain mediates an interaction with Cullins through a paired Cullin 2 (Cul2) and Cullin 5 (Cul5) interaction motif that overlaps with amino acids critical for transcription activation function [19, 20]. ZTA can also interact with p53 through its b-ZIP domain [21] and can target p53 for degradation through a mechanism that is dependent on the Zta-Cullin interaction [20]. In this context, ZTA has been shown to function as an adaptor in the Elongin B/C-Cul2/5-SOCS (ECS) ubiquitin ligase complex. EBV, like other herpesviruses, encodes several ubiquitin deconjugating enzymes [22], at least one of which, BPLF1, stabilizes ubiquitylated substrates necessary for EBV lytic cycle replication [23]. BPLF1 promotes viral DNA replication through inhibiting multiple Cullin-Ring-ubiquitin ligase (CRL), including those that ubiquitylate cellular S phase licensing factor CDT1 [24]. BPLF1 was shown to block the interaction of Cullin with the Cullin-associated and neddylation-dissociated 1 (CAND1) protein. CAND1 is an F-box exchange factor required for loading substrates onto the CRL complex required for ubiquitylation and proteasome degradation of numerous protein substrates [25]. CAND1 is thought to regulate the exchange of the Cullin-ROC1 module with different SKP1-F-box substrate complexes [26, 27]. CAND1 has been implicated as an inhibitor of viral replication since it competes with BPLF1 for interaction with Cullins [23]. How the ECS, CRL, and CAND1 exchange systems regulate EBV reactivation from latency is not completely understood.

Recent work from our lab identified a new class of small molecular activators of EBV lytic reactivation [28]. We identified several molecules with a common tetrahydrocarboline core that stimulated ZTA and EA-D expression in multiple different cell lines latently infected with EBV, including those derived from Burkitt lymphoma, nasopharyngeal carcinoma, and lymphoblastoid cell lines immortalized in vitro [28]. The most active molecule, C60, was found to have a mechanism of action distinct from HDAC inhibitors and phorbol esters. Here, we provide evidence that C60 works through a novel mechanism that stabilizes p53 and ZTA by altering CAND1-dependent Cullin-ubiquitin ligase substrate exchange and selection.

## Results

### Identification of CAND1 as a C60-affinity purified protein

**Fig 1:**
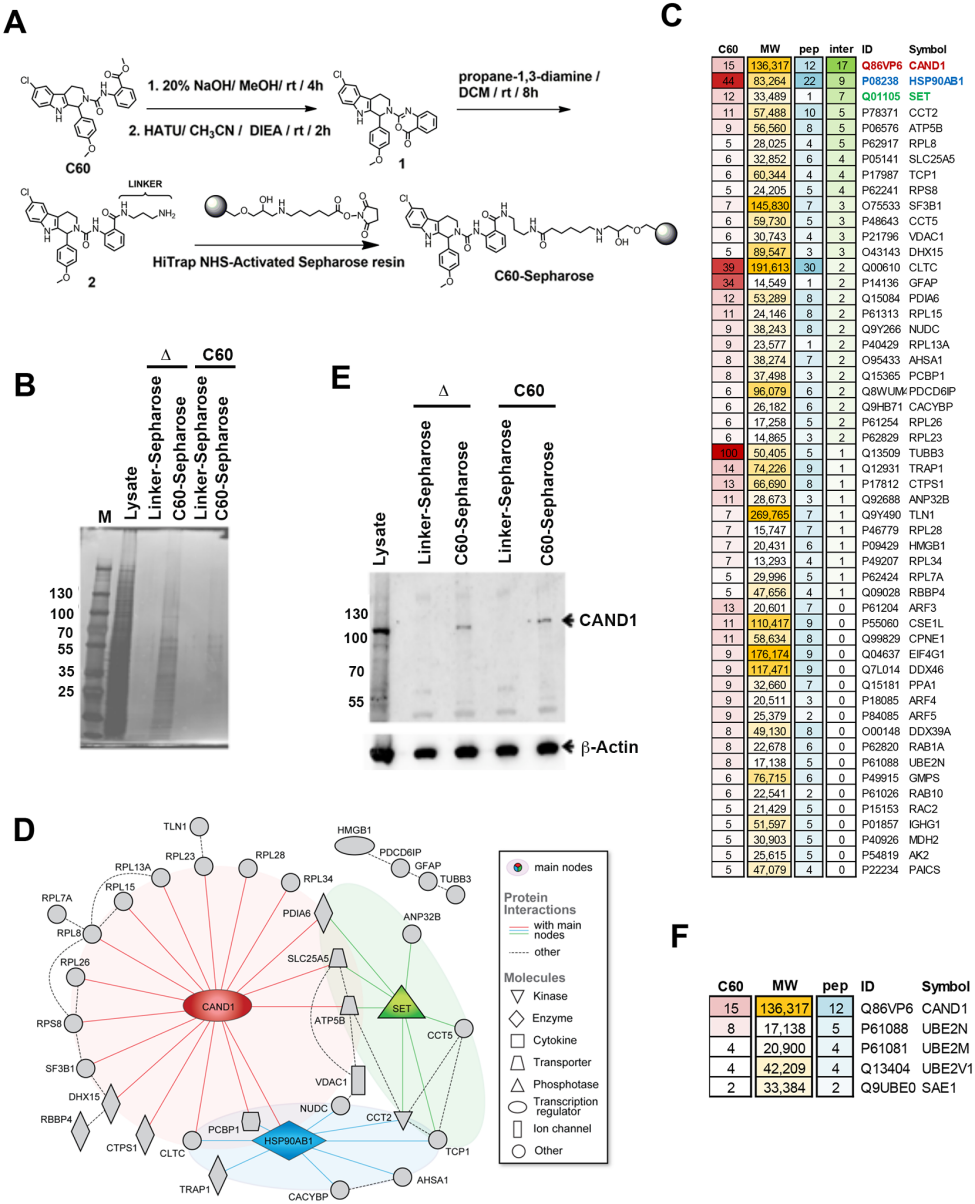
Identification of CAND1 protein network by C60-affinity purification. **(A)** Chemical structure of C60 and scheme for generating C60-sepharose using HiTrap NHS resin and an amide linker. **(B)** Silver stain of C60-affinity purification. Mutu I cell total cell extract (Lysate) was incubated with Linker-Sepharose or C60-Sepharose and eluted with SDS elution buffer and heat (Δ) or with 10 μM C60 (C60 elute). M is marker proteins with Kd indicated. **(C)** List of proteins identified by LC-MS/MS showing unique peptides in C60 eluted from C60 or Linker Control resin. Only proteins detected with at least 5 spectra counts in C60, but none in blank or control are shown. C60 = spectra counts for C60 sample. MW = molecular weight, pep = number of unique peptides, inter = total number of known protein-protein interactions with other proteins from the list. **(D)** Known protein-protein interactions among the list of most abundant proteins identified from LC-MS/MS C60 affinity purification shows CAND1 as the main hub of the identified network. **(E)** Western blot for proteins shown in panel B probed with anti-CAND1 (top panel), or anti-Actin (lower panel). **(F)** List of proteins involved with CAND1 in protein modification network (ubquitylation, sumoylation) identified in C60 affinity purification, but not shown in panel **C**.

To identify proteins that interact with C60, we chemically coupled C60 to HiTrap N-hydroxysuccinimide (NHS)-activated sepharose resin through an amide linker as shown in Fig 1A (see Methods for details). We then used C60-coupled resin or mono-N-Boc-propane-diamine-control linker-coupled resin to affinity purify proteins from Mutu I total cell extracts. Extracts were incubated, washed, and then eluted with either C60 or boiled in SDS-PAGE elution buffer (Δ), and then analyzed by SDS-PAGE followed by silver stain (Fig 1B). C60 eluted material was subject to LC/MS/MS which identified 136 proteins bound exclusively in the C60 affinity resin experiment with at least 2 spectral counts, but not in the linker resin or blank samples (S1 Table) with the top 52 proteins having 5 or more spectral counts (Fig 1C). Among the top hits that were bound exclusively in the C60 affinity resin and not the linker resin was CAND1. A proteome network analysis of the most abundant proteins recovered selectively on the C60 resin also identified CAND1 as a central hub in this network with the most known protein-protein interactions, which may explain the majority of hits bound in the C60 resin (Fig 1D). A second hub was centered around the SET protein, which shares common interaction partners with CAND1. We validated by Western blot that CAND1 was selectively enriched in the C60 affinity resin relative to the linker control resin (Fig 1E). We also found other proteins involved in a protein modification network (ubquitination, sumoylation) that are biologically linked to CAND1 function, including UBE2N, UBE2M and SAE1 (Fig 1F).

### Identification of CAND1 in an shRNA screen for EBV lytic cycle restriction factors

**Fig 2:**
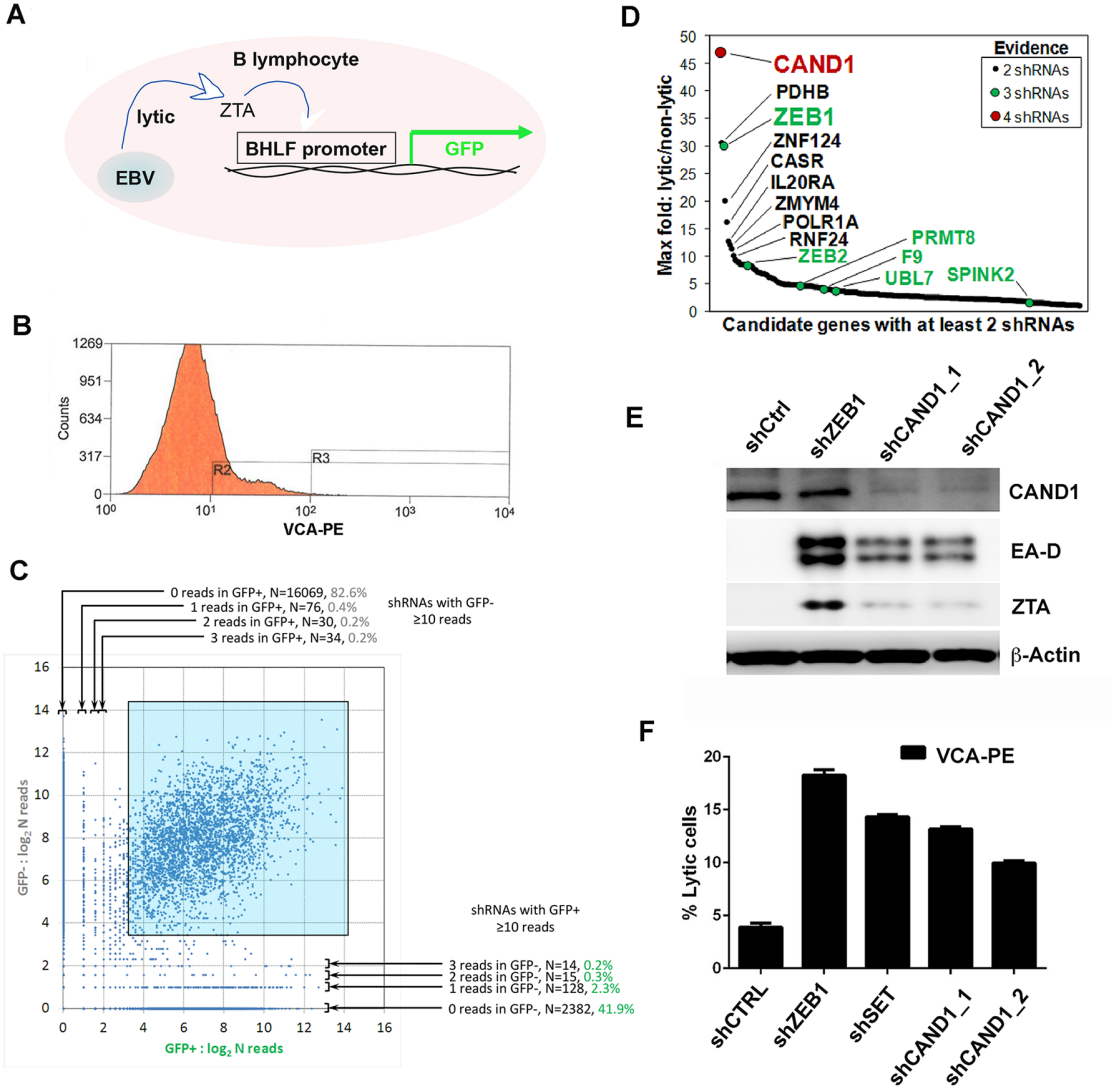
Identification of CAND1 by shRNA screen for lytic reactivation. **(A)** Mutu I cells containing BHLF1-GFP were transduced with shRNA lentivirus library and FACS sorted for GFP. **(B)** LCL cells were transduced with shRNA lentivirus library and FACS sorted for VCA. **(C)** Correlation of Illumina sequencing of shRNAs from GFP^+^ vs GFP^-^ populations for transduced Mutu I cells described in panel A. **(D)** Scoring of shRNA clones from combining both GFP^+^ Mutu I cells and VCA^+^ LCL cells after transduction with shRNA lentivirus library. Genes with 4 different targeting shRNA are indicated in red, 3 shRNA in green. Lytic vs non-lytic score reflects the percentage of shRNA found in each FACS sorted population. **(E)** Western blot analysis of Mutu I cells transduced with shCtrl, shZEB1, zhCAND1_1, or shCAND1_2, and probed with antibody to CAND1, viral antigens EA-D or ZTA, or β-Actin. **(F)** The percentage of EBV lytic cells was measured by FACS sorting for VCA^+^ Mutu I cells after transduction with shCTRL, shZEB1, shSET, shCAND1_1, or shCAND1_2.

In a parallel effort, we performed an shRNA screen to identify host target genes that restrict spontaneous lytic reactivation. EBV positive LCL or Mutu I cells carrying a stable episome with the EBV early lytic promoter BHLF1 driving GFP expression were transduced with the TRC lentivirus shRNA library [29], and sorted by FACS for Viral Capsid Antigen (VCA) or GFP (Fig 2A–2C). We then recovered shRNA DNA sequence by PCR from EBV reactivated cells relative to unsorted cells and processed the DNA for Illumina NextGen sequencing. Next, we scored reactivation hits from both cell lines and screening approaches for the cumulative number of different shRNA clones (Fig 2C). We found that CAND1 was the top hit based on the identification of 4 different shRNA targeting clones, while the next top hits, PDHB and ZEB1, recovered only 3 shRNA clones (Fig 2D). ZEB1 has previously been shown to be a constitutive repressor of EBV lytic reactivation [30, 31], providing validation for the shRNA screening method. We compared the ability of shCAND1 (clone_1 and clone_2) to reactivate EBV from transduced LCLs relative to shZEB1 (Fig 2E). Although shCAND1 was not as potent as shZEB1, it stimulated both Zta and EA-D expression relative to control shRNA (shCtrl) (Fig 2E). Similarly, shCAND1_1 and shCAND1_2 stimulated VCA expression levels similar to that of shZEB1 and shSET as measured by FACS (Fig 2F). These studies indicate that CAND1 is a restriction factor for EBV lytic reactivation from latency in Mutu I and LCLs.

### C60 induces a p53-stress response pathway distinct from HDAC inhibitor sodium butyrate

**Fig 3:**
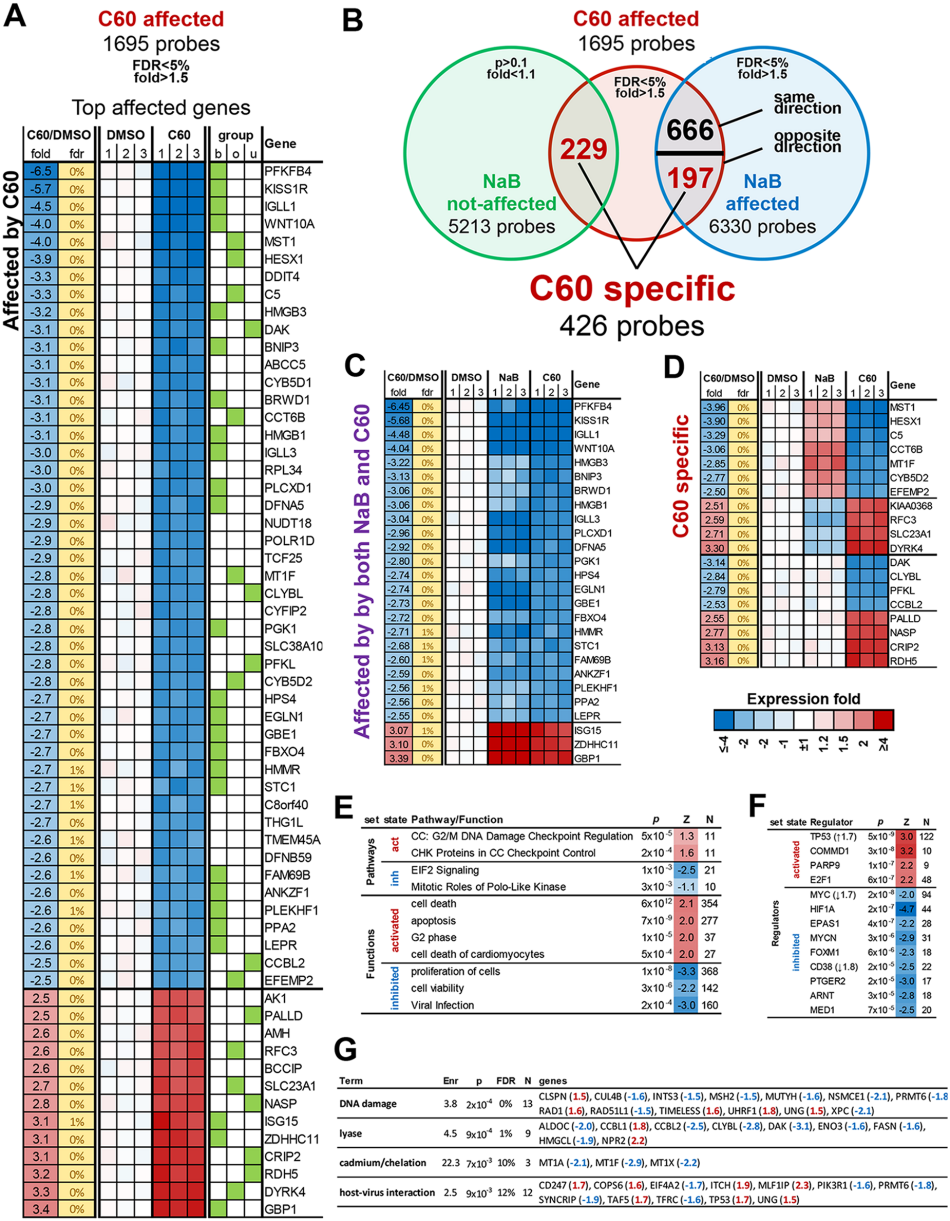
Gene expression profiling of C60 treated Mutu I cells. **(A)** RNA expression heatmap for genes affected >2.5 by C60 treatment. Group indicates if the gene was affected by b = both C60 and NaB, o = opposite by C60 compared to NaB and u = uniquely by C60. **(B)** Overlap between C60 and NaB transcriptional effect identifies sets of common and specific to C60 genes. **(C)** Genes affected by both C60 and NaB 2.5 fold or more. **(D)** Genes affected specifically by C60 2.5 fold or more in opposite or unique effect relative to NaB. **(E-F)** IPA analysis for regulators, pathways and functions enriched among genes significantly affected by C60. Z = z-score for predicted activation state of the category: positive = activated by C60, negative = inhibited by C60. Where significant, regulators are listed with mRNA level changes in parentheses. **(G)** DAVID analysis for swiss-prot categories significantly enriched among genes with a specific C60 effect (affected only by C60 or in opposite way compared to NaB) with genes fold changes listed in parenthesis. Enr = fold enrichment, FDR = false discovery rate.

To better understand the mechanism of action of C60, we interrogated its effect on cellular gene expression. We compared the mRNA expression profiles in Mutu I cells treated with either DMSO, C60, or sodium butyrate (NaB) using Illumina HumanHT-12V4 expression Beadchip. We found 1695 probes significantly affected by C60 (FDR<5%, at least 1.5 fold, S2 Table) with 60 known genes changed at least 2.5 fold (Fig 3A). While C60 and NaB had some overlapping target genes (666 probes, Fig 3B), among the most affected genes were repression of WNT10A and KISS1R and activation of GBP1 and ZDHHC11 (Fig 3C). There were many differences between these two treatments, including 229 genes uniquely affected by C60 and 197 genes affected in the opposite direction to NaB (top affected genes in Fig 3D), suggesting that their mechanism of action is different. This finding is consistent with our previous study indicating that C60 does not function as an HDAC inhibitor, since it did not increase global levels of histone acetylation [28]. Ingenuity Pathways Analysis indicated that C60 activates a G2/M DNA Damage Checkpoint and inhibits EIF2 Signaling, while increasing apoptosis and inhibiting proliferation and cell viability (Fig 3E). The Viral Infection pathways was also inhibited by C60 (Fig 3F). Ingenuity Regulator analysis suggested that C60 was inhibiting Myc, while activating the TP53 and CDKN1 pathways (Fig 3F). In addition, C60 showed a significant activation of targets of COMMD1, a known CAND1 competitor that interacts with multiple Cullins and prevents their binding with CAND1 [32], suggesting C60 functions through inhibition of CAND1 activity. We also analyzed genes with specific response to C60 for enrichment of general functional categories (Fig 3G). This analysis identified DNA Damage Response and Host-Virus Interaction as functional categories linked to C60.

### C60 induces p53 protein in multiple cell types independent of EBV

**Fig 4:**
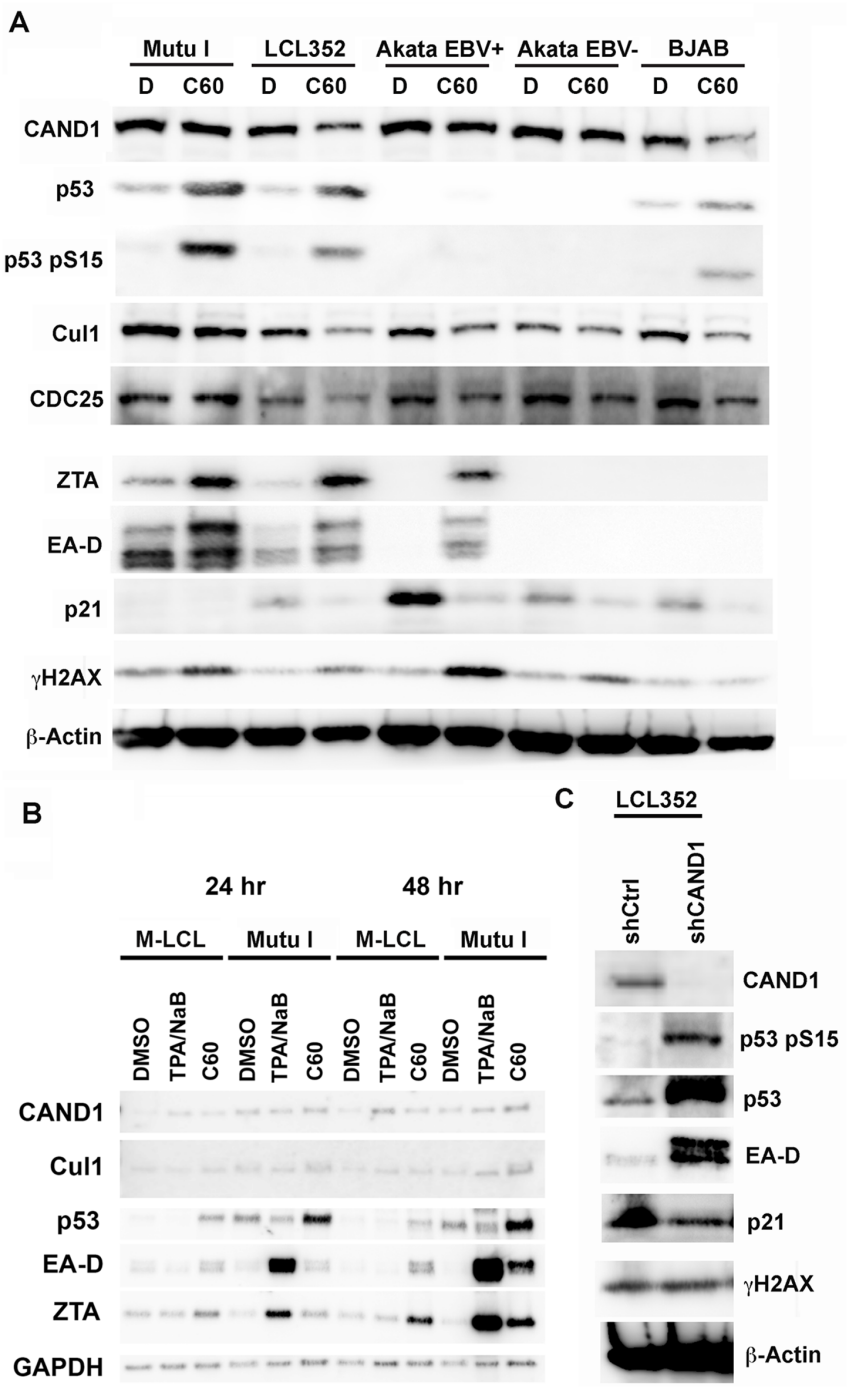
Stabilization of p53 and destabilization of p21 by C60. **(A)** Western blot of extracts from Mutu I, LCL352, Akata EBV^+^, Akata EBV^-^, and BJAB cells treated with DMSO (D) or C60 and probed with antibody to either CAND1, p53, p53 pS15, Cul1, CDC25, ZTA, EA-D, p21, *γ*H2AX, or β-Actin, as indicated. **(B)** Comparison of C60 with TPA/NaB relative to DMSO control at 24 and 48 hrs post treatment in Mutu-LCL (M-LCL) and Mutu I cells. **(C)** Western blot of LCL352 cells transduced with shCtrl or shCAND1_1 were probed with antibodies to CAND1, p53 S15, p53, EA-D, p21, *γ*H2AX, or β-Actin.

To further investigate the effect of C60 on the various pathways identified by functional genomics studies described above, we treated various EBV positive and negative B-cell lines with DMSO or C60 and assayed total cell lysates by Western blot with antibodies for CAND1, p53, phosphorylated p53 (pS15 p53), Cul1, CDC25, ZTA, EA-D, p21 (CDKN1A), *γ*H2AX, and β-Actin (Fig 4a). We found that C60 treatment had only minor effects on CAND1 and Cullin 1 levels, whereas it produced a significant activation of total p53 and pS15 phosphorylated p53. Although Akata cells lack detectable p53, they still showed EBV lytic reactivation in response to C60, indicating that p53 is not essential for viral reactivation by C60. EBV negative BJAB cells also had increased p53 in response to C60, indicating that EBV genes or DNA replication is not required for p53 activation by C60. As expected, there was an activation of EBV early antigens ZTA and EA-D. Interestingly, p21 expression, typically activated by p53, was reduced relative to DMSO treated cells, including Akata cells that lack detectable levels of p53. C60 weakly activated *γ*H2AX in most cells, but this was not observed in BJAB cells, suggesting that DNA damage is not the primary mode of action of C60.

To better understand the mechanism of action of C60, we compared its effects with that of TPA plus sodium butyrate (TPA/NaB) on Mutu-LCL (M-LCL) and Mutu I cells 24 and 48 hrs post-treatment (Fig 4B). As above, C60 had subtle effects on CAND1 and Cullin 1 expression. On the other hand, C60 increased p53 protein levels as early as 24 hrs after treatment in M-LCL and Mutu I cells, while TPA/NaB had only modest effects on p53 levels relative to DMSO control. TPA/NaB treatment led to robust activation of ZTA and EA-D in Mutu I cells by 24 hrs, but had little effect on M-LCL cells. In contrast, C60 induced ZTA and EA-D at 48 hrs in both Mutu I and M-LCL cells, but had little effect on ZTA and EA-D at 24 hrs (Fig 4B). These findings suggest that C60 works through a different mechanism than TPA/NaB, and that its effects on ZTA and EA-D are temporally delayed relative to its effects on p53. We also analyzed several of these proteins in LCLs after transduction with lentivirus shCAND1 (Fig 4C). We found that CAND1 depletion led to an increase in total and pS15 p53, and a decrease in p21 expression. CAND1 depletion induced EBV early antigens ZTA and EA-D as expected, but did not lead to a significant increase in *γ*H2AX. Taken together, these findings indicate that C60 increases levels of total and pS15 p53 and reduces the levels of its cell cycle arresting target p21. Furthermore, these effects are consistent with C60 phenocopying shRNA depletion of CAND1.

### C60 destabilizes CAND1-Cullin 1 interaction while increasing global ubiquitylation

**Fig 5:**
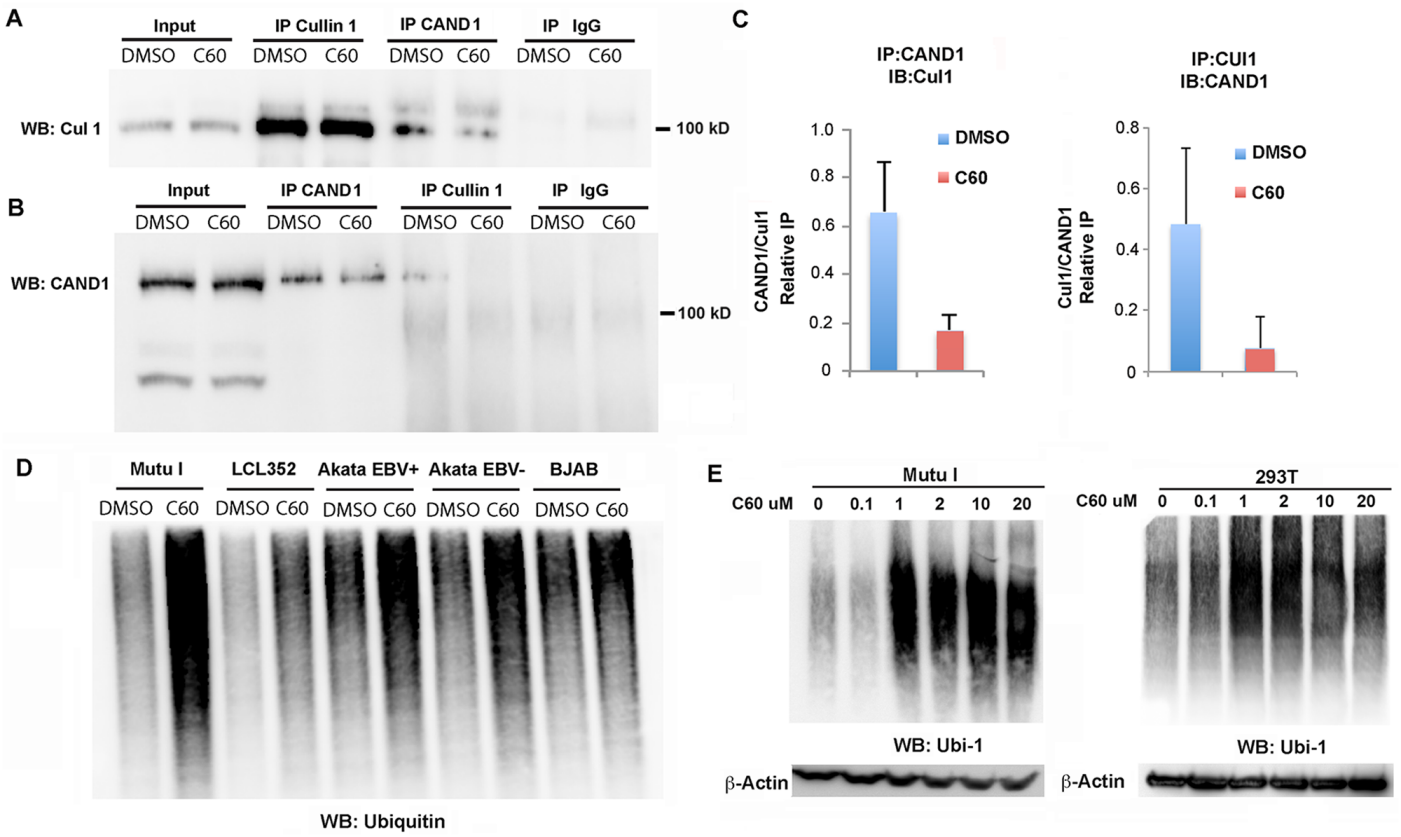
C60 disrupts CAND1-Cullin1 interaction and increases global ubiquitylation. **(A-C)** Extracts from Mutu I cells treated with DMSO or C60 for 48 hrs were subject to IP with antibody to Cullin 1, CAND1, or IgG and analyzed by Western blot for Cullin1 **(A)** or CAND1 **(B)**. Input (10%) is shown in first two lanes. **(C)** Quantitation of three independent coIP experiments as represented in panels A and B. **(D)** Mutu I, LCL352, Akata EBV^+^, Akata EBV^-^, and BJAB cells were treated with DMSO or C60 for 48 hrs, and then assayed by Western blot with antibody to Ubiquitin 1 (Ubi-1). **(E)** Dose dependent activation of global ubiquitylation in Mutu I (left) and EBV-negative 283T (right) cells. Total cell extracts were treated with indicated concentration of C60 for 48 hrs. Actin loading control is shown below.

CAND1 is known to interact with Cullin 1 [27]. Therefore, we tested whether C60 had an effect on this interaction (Fig 5A and 5B). Mutu I cells treated with DMSO or C60 were used to generate total cell lysates for IP with Cullin1 or CAND1 and probed by Western blot for Cullin 1 (Fig 5A) or CAND1 (Fig 5B). We found that C60 reduced the association of Cullin1 with CAND1 in both Cullin 1 IPs (Fig 5A) and CAND1 IPs (Fig 5B). Quantification of this interaction from several independent biological replicates indicates that C60 decreases CAND1-Cullin1 association by ~ 5 fold (Fig 5C and S1 Fig). These findings indicate that C60 destabilizes the interaction between Cullin1 and CAND1.

Since CAND1 and Cullins play a primary role in regulating protein ubiquitylation, we next asked whether the total abundance of ubiquitylated proteins was altered by C60 (Fig 5D). We found that C60 led to an increase in the accumulation of ubiquitylated proteins in all cell lines tested, including Mutu I, LCL352, Akata EBV+, Akata EBV-, and BJAB (Fig 5D). This effect was observed at concentrations as low as 1 μM in Mutu I cells, as well as EBV-negative 293T cells (Fig 5E).

### C60 functions through ZTA transcription activation domain

**Fig 6:**
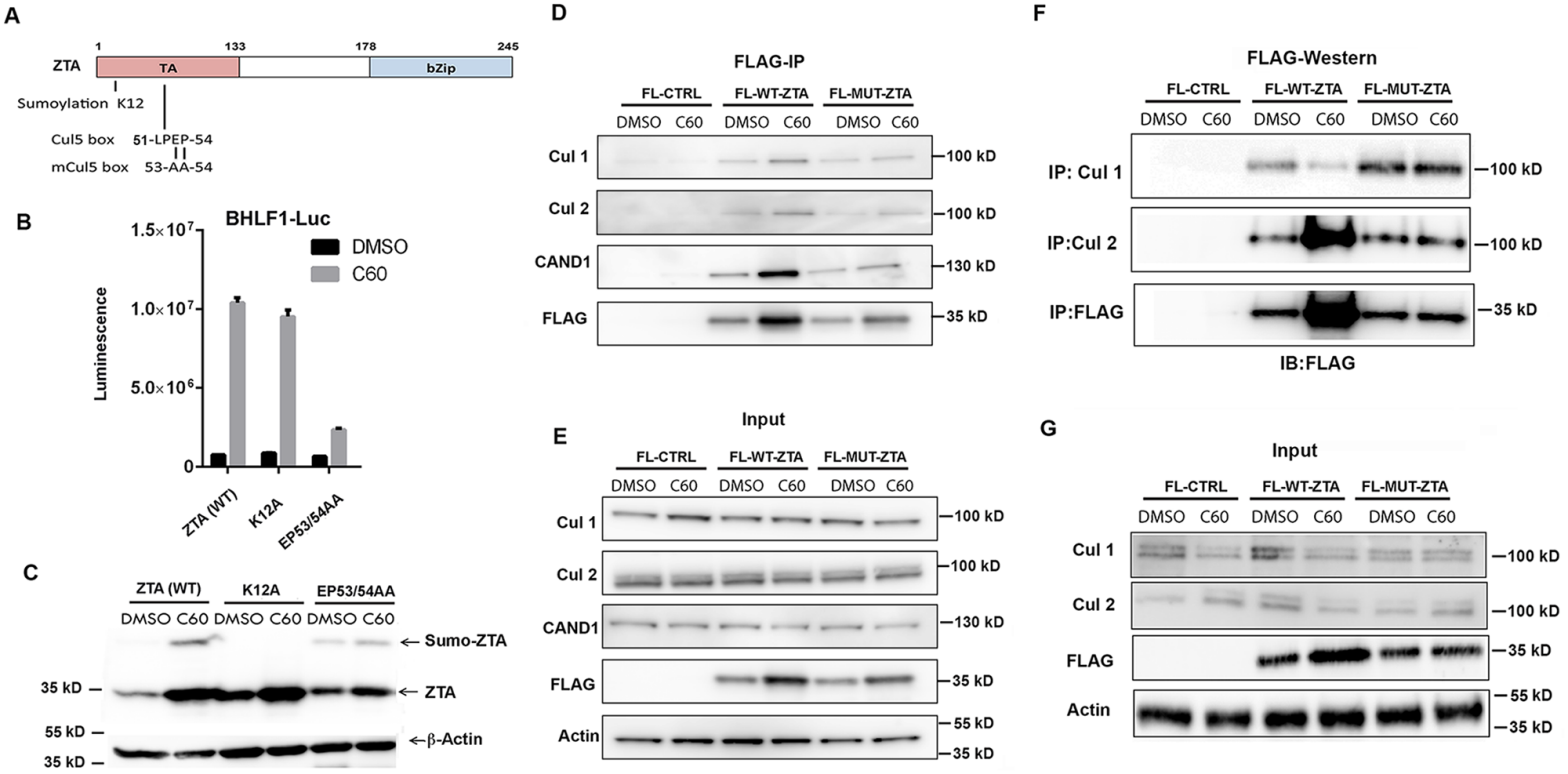
ZTA transcription activation domain mediates interactions with CAND1 and Cullins. **(A)** Schematic of ZTA transcription activation (TA) indicating position of sumoylation site at K12 and Cul5 box 52-LPEP-54. **(B)** Luciferase assay for BHLF1-Luc cotransfected with expression vector for ZTA (WT), K12A, or EP53/54AA (MUT) in 293T cells treated with DMSO (black bars) or C60 (grey bars) for 48 hrs. **(C)** Western blot of 293T cells transfected with ZTA (WT), K12A, or EP53/54AA treated with DMSO or C60 for 48 hrs, as described in panel B. **(D-E)** 293T cells transfected with FL-CTRL, FL-WT-ZTA, or FL-MUT-ZTA were treated with DMSO or C60 for 48 hrs, and then subject to IP with FLAG and probed with antibodies for Cul 1, Cul 2, CAND1, FLAG, or Actin **(D)**. 10% of input was analyzed by Western for Cul 1, Cul 2, CAND1, FLAG, and Actin **(E)**. **(F-G)** 293T cells were transfected as in D-E, and subject to IP with either Cul 1, Cul 2, or FLAG, and then analyzed by Western blot with antibody to FLAG **(F)**. 10% of input was analyzed by Western for Cul 1, Cul 2, FLAG, and Actin **(G)**.

To better understand how C60 may enhance EBV reactivation, we tested the effect of C60 on the EBV lytic switch transcriptional activating protein ZTA (Fig 6). ZTA has been reported to have a Cullin interaction motif (51-LPEP-54) in its transcriptional activation domain (Fig 6A) [20]. A mutation in this motif (EP53/54AA) was previously shown to reduce ZTA transcription activation function [19]. ZTA is also known to be sumoylated at K12 [17]. 293T cells were co-transfected with either wild type (WT) ZTA, K12A, or EP53/54AA and the BHLF1-Luc reporter followed by treatment with DMSO or C60 and analyzed by Luciferase assay (Fig 6B) or Western blot (Fig 6C). C60 induced Zta transcription activation function for ZTA (WT) and K12A, but was compromised for EP53/54AA (Fig 6B). We also observed that C60 increased ZTA WT and K12A protein stability, but this effect was compromised for EP53/54AA (Fig 6C). This suggests that the EP53/54AA mutation partially disrupts C60-dependent stabilization of ZTA.

### Zta activation domain mediates interaction with Cullins and CAND1

We next compared the ability of FLAG-ZTA WT (FL-ZTA-WT) and transcription activation defective mutant EP53/54AA (FLAG-ZTA-MUT) to co-immunoprecipitate with Cullin 1, Cullin 2, or CAND1. We found that FLAG-ZTA-WT co-precipitated with Cullin 1, Cullin 2, and CAND1, while FLAG-ZTA-MUT did not, suggesting that transcription activation function is linked to the interactions with Cullins and CAND1. We also examined the effect of C60 on the interaction of ZTA with these factors and found little effect by FLAG-IP, although C60 tended to restore FLAG-ZTA-MUT ability to interact with Cullins and CAND1 (Fig 6D and 6E). On the other hand, reverse IPs with Cullin 2 and Cullin 1 suggest that C60 increases the association of ZTA-WT with Cullin 2, and decrease its interaction with Cullin 1 (Fig 6F and 6G). These latter results may suggest that C60 alters the relative affinity of ZTA for different Cullins.

### Ubiquitin-proteolysis regulates ZTA protein stability and EBV reactivation pathway

**Fig 7:**
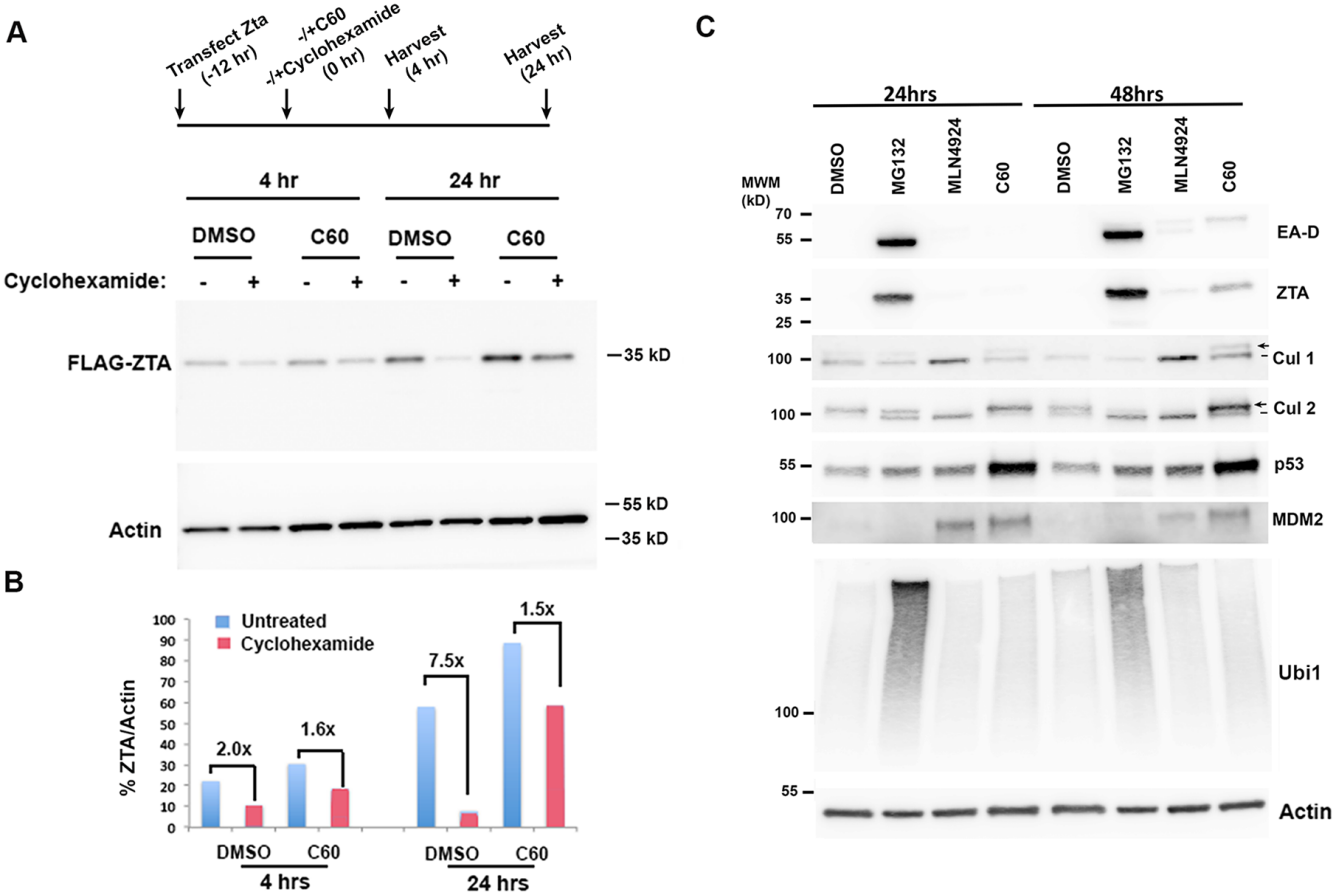
C60 stabilizes ZTA through a mechanism distinct from MG132 and MLN4924. **(A)** Timeline of ZTA protein stabilization assay with addition of cyclohexamide (20 μg/ml) in the presence of either C60 (1 μM) or DMSO at 12 hrs post-transfection followed by cell collection at 4 and 24 hrs after addition of cyclohexamide. Western blot for FLAG-ZTA and Actin in 293T cells transfected and treated as shown in timeline above. **(B)** Quantification of Western blot shown in panel A as FLAG-ZTA relative to Actin at each time point. **(C)** Mutu I cells treated with DMSO, MG132 (20 μM), MLN4924 (0.5 μM), or C60 (5 μM) were assayed by Western blot at 24 hrs or 48 hrs after drug treatment. Western blots were probed with antibodies to EA-D, ZTA, Cul 1, Cul 2, p53, MDM2, Ubiquitin (Ubi-1) and Actin, as indicated.

Our findings suggest that C60 induces EBV reactivation through modulation of the ubiquitin proteolysis pathways. To assess ZTA protein stability, we treated FLAG-ZTA transfected 293T cells with cyclohexamide at 12 hrs post-transfection to block any additional protein synthesis. We then measured ZTA protein levels at 4 and 24 hrs after cyclohexamide treatment in cells treated with C60 or DMSO control (Fig 7A and 7B). We found that ZTA protein degraded 7.5 fold relative to Actin by 24 hrs post-cyclohexamide treatment in DMSO control treated cells. In contrast, ZTA protein degraded only 1.5 fold in C60 treated cells at the same time point, providing ~3.3 fold increase in protein stability relative to DMSO. This suggests that C60 functions, at least in part, at the level of ZTA protein stabilization. To investigate further the role of ubiquitin proteolysis in regulating ZTA stabilization and EBV reactivation from latency, we compared C60 to two other small molecules with well-characterized targets in the ubiquitin-conjugating and proteolysis pathway. We compared the effects of C60 with the proteasome inhibitor MG132 and the NEDD8 Activating Enzyme (NAE) inhibitor MLN4924 for their ability to induce ZTA and EA-D in Mutu I BL cells (Fig 7C). As before (see Fig 4), we observed that C60 stabilized p53 at 24 and 48 hrs post treatment, while ZTA and EA-D were detected at 48 hrs post-treatment (Fig 7C). NAE inhibitor MLN4924 had only a minor activating effect on ZTA and EA-D protein levels, but its activity could be detected by the accumulation of a faster migrating deneddylated isoform of Cullins 1 and 2 (Fig 7C). Interestingly, C60 resulted in the accumulation of the slower migrating, presumably Neddylated, form of Cullins 1 and 2. Also striking, was the rapid effect of MG132 on abundance of ZTA and EA-D at 24 and 48 hrs. Taken together, these data suggest that C60 functions in the Cullin-Neddylation pathway, but through different mechanisms than MLN4924 and MG132. These results also indicate that ZTA and EBV lytic reactivation control are highly sensitive to proteasome-dependent control pathways.

## Discussion

**Fig 8:**
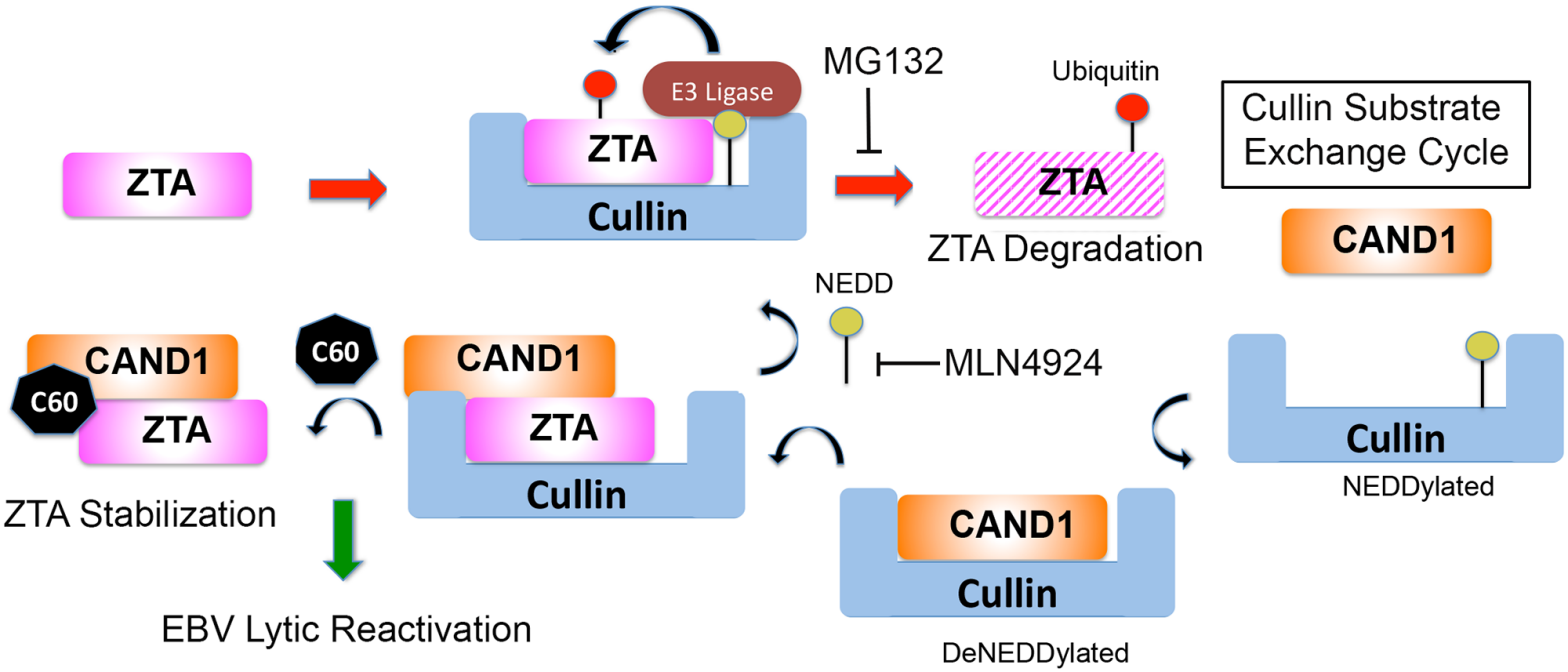
Model of C60 perturbation of CAND1-Cullin function in destabilizing ZTA and p53. CAND1 recycles Cullins to generate new CRLs that degrade ZTA (right red arrow). C60 alters CAND1 interaction with Cullins to prevents ubiquitin-mediated degradation of ZTA, resulting in EBV reactivation from latency. C60 works through a different arm of the pathway than proteasome inhibitor MG132 or NAE inhibitor MLN4924.

Strategies to reactivate EBV from latency are of current clinical interest for precision treatment of cancers latently infected with EBV [5, 7, 12, 33]. We previously identified the small molecule C60 as a potent activator of EBV reactivation from various latently infected cell types [28]. We now show through biochemical affinity purification that C60 can interact with the cellular protein CAND1 (Fig 1). By genetic shRNA library screening, we demonstrated that CAND1 acts as a restriction factor for EBV reactivation from latency (Fig 2). Gene expression profiling data revealed that C60 affects a spectrum of gene targets distinct from the HDAC inhibitor NaB, and targets pathways linked to p53 and cell cycle control (Fig 3). We found that C60 induces p53 phosphorylation and protein stability at early time points, while activating ZTA and EA-D at subsequent times (Fig 4). C60 inhibited the interaction between CAND1 and Cullin1, and increased the global cellular accumulation of ubiquitylated proteins (Fig 5). In terms of EBV gene regulation, we found that C60 stabilized the immediate early protein ZTA through a mechanism that was partly dependent on functional residues within the ZTA transcriptional activation domain and important for interaction with Cullins and CAND1 (Fig 6). Finally, we demonstrate the ubiquitin-dependent proteasome regulation controls ZTA and EA-D levels, and that C60 works through a mechanism distinct from proteasome inhibitor MG132 and Neddylase inhibitor MNL4924 (Fig 7). Taken together, we propose a model (Fig 8) where C60 perturbs the interaction between CAND1 and Cullins to alter the ubiquitylation cycle of critical targets, such as p53 and ZTA, that regulate EBV lytic reactivation from latency.

### CAND1, Cullins, and ubiquitylation

CAND1 is known to interact with Cullins and prevent their reassembly as functional CRLs with new substrates [34]. CAND1 binding to Cullin 1 is regulated by Nedd8 covalent modification of Cullin 1 [35]. Cullins undergo a Neddylation cycle for their activation, and CAND1 is known to interact with deneddylated Cullins and facilitate F-box receptor-substrate exchange. CAND1 stimulates ubiquitylation of some substrates by facilitating the exchange of Cullins with different CRL adaptor-substrate modules [36]. Hypothetical modulators of CAND1 are predicted to have cell-type and context-dependent effects on different CRLs and their ubiquitin substrates [25]. Our data indicates that C60 perturbs CAND1 interaction with Cullin 1. How this confers greater stability to p53 and ZTA is not completely understood. We propose that C60 inhibits the ability of CAND1 to exchange Cullin-p53 or Cullin-ZTA substrates, resulting in the failure of these substrates to get ubiquitylated and degraded (Fig 8). On the other hand, C60 may accelerate the ubiquitylation and degradation of other substrates, since we observed a global increase in ubiquitylation and a reduction of some proteins, such as p21. The F-box adaptors and CRLs involved in p21 have been identified [37–40], and may represent a different class of CRLs than those responsible for the degradation of ZTA and p53. Future studies will be needed to determine how C60 affects these substrate-specific CRLs.

### Transcriptional activation function and CAND1 regulation

The relationship between ubiquitylation and transcription activation has been well-documented [41, 42]. CAND1 was initially identified as the TBP-Interacting Protein 120A (TIP120A) [43, 44], suggesting it may contribute directly to the transcription activation domain function of proteins like p53 and ZTA. Ubiquitylation of transcription activation domains is known to play an integral role in the transcription activation cycle [41]. Many transcriptional activation domains (TADs), including that of p53 and ZTA, contain degrons that target ubiquitin mediated degradation [41]. Previous studies revealed that amino acids 53E and 54P in ZTA TAD are critical for transcription activation function [19], and more recent studies revealed that these overlap with a consensus Cul5-interaction motif [20]. ZTA was found to function as an adaptor for Cul2 and Cul5- containing ECS (Elongin B/C-Cul2/5-SOCS- box protein) ubiquitin ligase to stimulate the ubiquitylation and degradation of p53 [20]. We found that ZTA can also interact efficiently with CAND1, Cullins 1 and 2, and that C60 can alter ZTA stability (Figs 6 and 7). Mutations EP53/54AA that reduce ZTA transcription activation, compromise the interaction with CAND1. These results suggest that the ZTA TAD function is regulated by specific CAND1 and CRL interactions that can be perturbed by C60. We suggest that C60 inhibits CAND1 function in the formation of new CRL complexes that degrade ZTA and p53, consequently enhancing their ability to activate transcription of downstream genes important for EBV reactivation.

### Regulation of EBV by CAND1-Cullin and C60

Other modulators of the CAND1-Cullin pathway have been found to alter EBV lytic replication. The EBV encoded tegument protein BPLF1 interferes with NEDD8 binding to Cullins, which stimulated EBV lytic replication [23]. It is possible that C60 mimics some of the activities of BPLF1. CAND1 has also been reported to be a target of miR148-b [45], which has been implicated in EBV gene regulation [46]. Recent genetic studies have also implicated CAND1 as a potential regulator of EBV copy number during EBV latency [47]. Our findings suggest that C60 targets CAND1 and alters its function in recycling Cullins that degrade ZTA and p53. However, C60 appears to act differently than NAE inhibitor MLN4924 that blocks Cullin neddylation and new CRL formation [48]. Unlike C60, MLN4924 did not did not stabilize ZTA and EA-D (Fig 7). On the other hand, proteasome inhibitor MG132 had a robust and rapid effect on ZTA and EA-D levels in Mutu I cells, suggesting that these proteins are regulated by ubiquitin-mediated proteolysis. The effect of C60 on ZTA and EA-D is likely to be partly indirect, as the effect is kinetically delayed compared to p53. However, this could be due to the slow accumulation of ZTA through several rounds of auto-activation. C60 may also induce p53 through an ATM-dependent stress pathway, such as DNA damage. However, C60 does not induce DNA-damage associated *γ*H2AX to levels approaching that of known DNA damaging agents, such as doxorubicin (S3 Fig), and doxorubicin does not activate EBV in all cell types as does C60. Thus, C60 is not likely to be a direct DNA damaging agent even though it can activate ATM signaling. We propose that C60 alters CAND1-Cullins to alter substrate selection for ubiquitylation and degradation. In conclusion, our findings suggest that the CAND1-Cullin pathway is an important regulatory node for EBV reactivation from latency, and can be manipulated by biological and pharmacological agents to potentially treat EBV associated disease.

## Materials and methods

### Cell culture

Akata (EBV^+^ BL), Akata EBV negative (EBV^-^ BL), Mutu I (EBV^+^ BL) were obtained from Jeff Sample (Penn State University, Hershey PA), BJAB, DG75 (EBV^−^BL), and 293T cells were obtained from ATCC. LCL352 or LCL187 (Mutu-LCL) were generated at the Wistar Institute by in vitro immortalization of human B-lymphocytes from EBV negative donors with the Mutu I strain of EBV. All cell lines were used at low passage and were cultured for no more than 1 month in RPMI supplemented with 10% heat inactivated FBS, 50 ng/ml penicillin, and 1% Glutamax (Invitrogen) at 37°C and 5% CO_2_. Cell concentration was maintained at 0.2–0.8 million per ml, and cell viability was over 90% for each cell line at the time of each treatment. Where indicated, cells were treated with Sodium Butyrate (1mM) / 12-*O*-tetradecanoylphorbol-13-acetate (TPA, 20μg/ml), MLN4924 (0.5μM), MG132 (20μM), and C60 (1 or 5μM, as indicated).

### Preparation of C60 affinity resin

The C60 ester was hydrolyzed to the carboxylic acid by stirring a solution of C60 in MeOH in the presence of 20% NaOH at room temperature for 4 hours. The resulting carboxylic acid was then dissolved in acetonitrile and treated with hydroxybenzotriazole (HATU) to generate the activated ester, which spontaneously cyclized to form intermediate **1** (Fig 1A). Treatment of **1** with propane-1,3-diamine in dichloromethane at room temperature for 8 hours provided the intermediate amide **2**, which provided an amine functional group that can be used for conjugation to HiTrap NHS-activated sepharose resin. We also treated intermediate **1** with the tert-butyl (3-aminopropyl)carbamate in dichloromethane at room temperature for 8 hours, to generate a neutral amide-linker as the N-Boc protected amine derivative of C60. This neutral N-Boc protected derivative of **2** efficiently stimulated EBV reactivation (IC50<500 nM), confirming that addition of a neutral linker did not adversely affect the affinity of the C60 ligand for our affinity pull down experiment. The amine, **2**, was then coupled to the HiTrap NHS-activated sepharose resin following a slightly modified procedure based on the technical manual provided by the vendor. The contents of a HiTrap NHS-activated HP prepacked 1 mL column was removed and added to a 5 mL polypropylene chromatography syringe barrel containing a fine frit. The material was washed with 1 mL of HPLC grade acetonitrile, then the syringe barrel was capped at the bottom and 1 mL of a 2 mg/mL solution containing amine **2** and 1% diisopropylethylamine (DIEA) was added to the NHS activated Sepharose resin (10 μmol NHS per 1 mL column of resin). The mixture was reacted for 5 hours with gentle mixing, then the bottle cap was removed and the resin was washed consecutively with water and acetonitrile. To react any remaining NHS groups the resin was treated with three consecutive washes with a 2% ethanolamine solution in water. The resin was then washed three times with 5 mL of a 0.5 M solution of sodium chloride, followed by three washes with 5 mL a 0.5 M solution of sodium acetate, followed by three washes with 5 mL of a 0.5 M sodium chloride solution. This material was then used for affinity based chromatography purifications. In addition to C60, we generated a control linker resin (mono-N-Boc propane-1,3-diamine) to help distinguish non-specific binding proteins from the C60 specific binding proteins.

### C60 affinity purification and proteomics

Mutu I cells were lysed in RIPA buffer (150 mM NaCl, 1.0% IGEPAL CA-630, 0.5% sodium deoxycholate, 0.1% SDS, and 50 mM Tris, pH 8.0) for 15 minutes at 4°C. Lysates were cleared from cell debris by centrifugation at 4000 rpm in an Eppendorf 5810R benchtop centrifuge at 4°C and passed through a low binding .45 μM filter (Millipore). Target identification was carried out by first passing the cleared cell lysate through an affinity column prepared by covalently attaching C60 to sepharose beads, followed by washing the column with RIPA buffer and phosphate-buffered saline (PBS) and eluting the C60-binding proteins with free C60 in PBS. Isolated proteins were separated on a 2D NuPage gel (Thermo) for analysis and visualized with Zinc Reversible Stain Kit (Pierce, cat# 24582). Eluate containing C60-binding proteins was trypsinized and analyzed by mass spectrometry at the Wistar Institute Proteomics Core facility.

### Proteomic analysis

Only proteins that were not detected in blank or linker-control experimental samples with at least 5 spectra counts were considered. Protein set enrichment analysis was done using QIAGEN’s Ingenuity Pathway Analysis software (IPA, QIAGEN Redwood City,www.qiagen.com/ingenuity) and “Biological function” and “Canonical Pathways” results were considered. Significance of enrichment was defined at nominal p-value<0.05. Only functions with predicted activation state (Z>2) were considered. Only pathways with at least 2 protein hits were considered. Protein-protein interactions were derived from Ingenuity Knowledgebase and gene network was generated including genes with at least one known interaction.

### Immunoblotting

Protein extract in Laemmli Buffer equivalent to 10^5^ cells were loaded on 8–16% SDS-PAGE gels (Invitrogen) and transferred to nitrocellulose membranes (Millipore). West Femto (Thermo) and ECL Prime (GE) were used to detect the proteins with Fujifilm LAS-3000 camera and software.

### Antibodies

Mouse anti-FLAG (cat# F1804), mouse anti-Actin (cat# 3854), and rabbit anti-CAND1were purchased from Sigma-Aldrich. Nonspecific rabbit IgG (sc-2027) and goat anti-CAND1 (A-13, cat# sc-10672) were purchased from Santa Cruz Biotechnology. Anti-Cul2 was purchased from Thermo (cat# 51–1800). Rabbit anti-Cul1 (cat# 4995), anti-p21 (cat# 2947), anti-p27 (cat# 2552), anti-CAND1 (cat# 7433), and mouse anti-p53 (cat# 2524), anti-phospho-p53Ser15 (cat# 9286), Cdc25A (cat# 3652) antibodies were purchased from Cell Signaling Technology. Anti-ubiquitin antibody anti-Ubi-1 (cat# ab7254) and anti-Cul1 (cat# ab78517) were purchased from Abcam. Anti-MDM2 (Ab-2) was mouse mAb (2A10) from Calbiochmem (Millipore) (cat# OP115). Rabbit polyclonal anti-ZTA was generated from full length Zta at Pocono Rabbit Farms, anti-VCA (Thermo, cat# MA1-7274) and anti-EA-D (Abcam, cat# ab49668) were described previously [28].

### Immunoprecipitation

IP was performed as described previously [20] with minor modifications. Cells were lysed in SDS-free lysis buffer (50 mM Tris-HCl pH 7.6, 120 mM NaCl, 0.1% NP40, 1 mM EDTA, 100 mM sodium fluoride, 2 mM sodium vanadate) for 15 minutes at 4°C with agitation. Lysates were centrifuged for 5 min at 4,000 rpm at 4°C to remove cell debris. Protein A-beads were precoupled with either nonspecific rabbit or mouse IgG or 10 μg specific antibody by incubating the mixture overnight at 4°C with agitation. Alternatively, pre-coupled Anti-Flag M2 agarose beads (Sigma-Aldrich) were added to cell lysates and incubated overnight at 4°C with agitation. The beads were washed in SDS-free lysis buffer three times, added to cell lysates and incubated overnight at 4°C with agitation. The beads were washed in PBS three times, lysed in Laemmli Buffer, boiled for 10 minutes and centrifuged at 14,000 rpm for 1 minute. Supernatants were transferred in the new tubes and 1/5 was loaded in each well for immunoblot analysis.

### FACS analysis and immunofluorescence

For the immunofluorescence staining of viral capsid antigen, cells were fixed with 4% paraformaldehyde in PBS, washed and blocked with PBS containing 1% BSA. Anti-VCA antibody (Millipore) was used at 1:100 followed by anti-mouse R-phycoerythrin (PE)-conjugated antibody (Sigma). FACS analysis was used to analyze the percentage of PE-positive cells. LSR-II instrument and FACSDiva software (BD Biosciences) were used to analyze the samples.

### Luciferase assays

Luciferase assays were performed as described in [28].

### High-throughput shRNA screening

4.5x10^6^ Mutu I or LCL352 cells were infected with TRC Lentiviral Library at MOI of 0.3 and cultured in RPMI supplemented with 15% FBS. Puromycin was added to cell culture 24 hrs after the infection. Samples were taken prior to infection, 24 hrs post infection before puromycin addition and 7 days after infection (6 days in puromycin selection). Genomic DNA was isolated with Quick-gDNA MiniPrep kit (Zymo) and used as a template to amplify shRNA fragments with custom primers (3’ ccttcaccgagggcctatttccc and 5’ actgccatttgtctcgaggtcgag). 100 nucleotides were sequenced from each end of the PCR fragment. WL (whole library), p3 (latent), p4 (marginal: lytic_latent) and p5 (the most lytic) samples were sequenced. Every read was tested for containing valid shRNA sequence and ~8.4M such reads per sample were obtained and then matched to library shRNA (~71% matched perfectly). Number of reads per each shRNA was then normalized to the ratio “maximum reads across all samples / reads in the sample.” For each shRNA, a normalize counts fold p5/max(p3,p4,WL) was calculated to find shRNAs enriched in the most lytic stage. Each gene was then annotated with number of shRNA for the gene that showed fold > 1 and maximum fold. Only genes with at least two shRNAs were considered and the data was plotted with genes with maximum fold > 10 or genes with at least 3 shRNAs were highlighted.

### shRNA-mediated knockdown of CAND1

Mutu I and LCL352 cells were infected with shRNA-carrying lentivirus and then selected with puromycin for 6 days as described above. All shRNA lentivirus constructs were from the Wistar screening facility’s TRC library. pLKO.1 vector-based shRNA construct for CAND1_1 (TRCN0000003459) and CAND1_2 (TRCN0000003460) were prepared for cell transductions as described previously [49].

### Plasmids and transfections

ZTA wild type and mutant constructs were described previously [19]. pHEBO-Hp- Luc was described in [28]. Transfections of 293T cells with Lipofectamine 2000 were performed following the standard protocol for Lipofectamine 2000 in 100mm plates (Invitrogen). Lipofectamine-DNA complexes were kept with cells overnight in OPTI-MEM medium (Invitrogen), then washed in PBS and kept in RPMI supplemented with 10% heat inactivated FBS, 50ng/ml penicillin, and 1% Glutamax (Invitrogen). Transfected cells were treated with either C60 or DMSO for 48–72 hours. Cyclohexamide was used at 20 μg/ml.

### mRNA expression profiling

Mutu I cells were maintained at low passage in RPMI containing 10% FBS as described above. 3x10^6^ cells were transferred to RPMI containing 5% FBS and treated with C60 or DMSO for 24 hours. The treatment was done in triplicate. 5x10^6^ cells were collected and washed twice in PBS. RNA was isolated with Trizol (Sigma) and resuspended in Tris-EDTA buffer. Total RNA at 100ng was amplified with Epicentre (cat# TAN07924) TargetAmp(tm) Nano-g(tm) Biotin-aRNA Labeling Kit to generate biotinylated, amplified RNA. Biotin labeled aRNA at 750ng was hybridized to an Illumina HumanHT-12V4 expression Beadchip using Illumina HumanHT-12 v4 Expression BeadChip Kit (cat# BD-103-0204). Subsequent steps include washing, blocking, and streptavadin-Cy3 staining of the beadchip. Fluorescence emission by Cy3 is quantitatively detected and GenomeStudio software provides results in standard file format. Signal intensity data was quantile normalized and genes that showed insignificant detection p-value (p>0.05) in all samples were removed from further analysis. Expression level comparisons between two groups was done using two sample t-test and correction for multiple testing to estimate False Discovery Rate (FDR) as described [50]. Genes with FDR<5% changed at least 1.5 fold were considered significant. Gene set enrichment analysis was done using QIAGEN’s Ingenuity Pathway Analysis software (IPA, QIAGEN Redwood City,www.qiagen.com/ingenuity) and “Biological Function,” “Canonical Pathways,” and “Upstream Regulators” results were considered with p-values and prediction of activation Z-scores used to filter results. Significance of enrichment was defined at nominal p-value<10^−3^, Z>2 for non-cancer related functions, p<10^−4^, Z>2 for regulators and FDR<15% Z>1 for pathways. DAVID enrichment analysis [51] was done considering only swiss-prot functional categories that pass thresholds of FDR<15%, enrichment>2 fold.

## Supporting information

S1 Fig Quantification of biological replicates for CAND1-Cul1 coIPs. Total cell lysates from Mutu I cells treated with DMSO or 1 μM C60 were subject to IP with antibody to Cul 1, CAND1, or control IgG, and then assayed by Western blot for Cul 1 (panel **A**), or CAND1 (panel **C**). Blots for 4 independent biological replicates (Expt 1–4) are shown. Quantitative densitometry is shown for each blot as intensity of Cul 1 in IP CAND1 relative to IP Cul 1 for (panel **B**) or for CAND1 in IP Cul 1 relative to IP CAND1 (panel **D**) each experiment 1–4.
(TIF)

S2 Fig C60 increases transcription of EBV latent, as well as lytic genes. RT-qPCR analysis for EBV gene transcription (as indicated) in Mutu I cells treated with either DMSO (black), or 5 μM C60 (orange) for 48 hours.
(TIF)

S3 Fig Comparison of C60 with doxorubicin for induction of DNA damage associated *γ*H2AX and p53 pS15 phosphorylation. LCLs were treated with 2 μM doxorubicin for 6 hrs, or 5 μM C60 for 24 of 48 hrs and assayed by Western blot for total p53, p53 pS15, *γ*H2AX, or GAPDH.
(TIF)

S1 Table List of proteins identified by LC/MS/MS with C60 affinity purification, normalized to linker-control. (XLSX)

S2 Table List of mRNA induced by C60 relative to NaB and DMSO controls in Mutu I cells. (XLSX)

## References

[1] Young LS, Rickinson AB. Epstein-Barr virus: 40 years on. Nature reviews Cancer. 2004;4(10):757–68. doi: 10.1038/nrc1452 . 15510157 10.1038/nrc1452

[2] Elgui de Oliveira D, Muller-Coan BG, Pagano JS. Viral Carcinogenesis Beyond Malignant Transformation: EBV in the Progression of Human Cancers. Trends in microbiology. 2016;24(8):649–64. doi: 10.1016/j.tim.2016.03.008 . 27068530 10.1016/j.tim.2016.03.008 PMC5489061

[3] Cohen JI, Mocarski ES, Raab-Traub N, Corey L, Nabel GJ. The need and challenges for development of an Epstein-Barr virus vaccine. Vaccine. 2013;31 Suppl 2:B194–6. doi: 10.1016/j.vaccine.2012.09.041 ; 23598481 10.1016/j.vaccine.2012.09.041 PMC3636506

[4] Young LS, Yap LF, Murray PG. Epstein-Barr virus: more than 50 years old and still providing surprises. Nat Rev Cancer. 2016 doi: 10.1038/nrc.2016.92 . 27687982 10.1038/nrc.2016.92

[5] Kenney S. Theodore E. Woodward Award: development of novel, EBV-targeted therapies for EBV-positive tumors. Transactions of the American Clinical and Climatological Association. 2006;117:55–73; discussion -4. ; 18528464 PMC1500921

[6] Israel BF, Kenney SC. Virally targeted therapies for EBV-associated malignancies. Oncogene. 2003;22(33):5122–30. doi: 10.1038/sj.onc.1206548 . 12910249 10.1038/sj.onc.1206548

[7] Lee HG, Kim H, Kim EJ, Park PG, Dong SM, Choi TH, et al Targeted therapy for Epstein-Barr virus-associated gastric carcinoma using low-dose gemcitabine-induced lytic activation. Oncotarget. 2015;6(31):31018–29. doi: 10.18632/oncotarget.5041 ; 26427042 10.18632/oncotarget.5041 PMC4741585

[8] Ambinder RF, Robertson KD, Moore SM, Yang J. Epstein-Barr virus as a therapeutic target in Hodgkin's disease and nasopharyngeal carcinoma. Seminars in cancer biology. 1996;7(4):217–26. doi: 10.1006/scbi.1996.0029 . 8946606 10.1006/scbi.1996.0029

[9] Gottschalk S, Rooney CM. Adoptive T-Cell Immunotherapy. Curr Top Microbiol Immunol. 2015;391:427–54. doi: 10.1007/978-3-319-22834-1_15 ; 26428384 10.1007/978-3-319-22834-1_15 PMC4655436

[10] McKenzie J, El-Guindy A. Epstein-Barr Virus Lytic Cycle Reactivation. Curr Top Microbiol Immunol. 2015;391:237–61. doi: 10.1007/978-3-319-22834-1_8 . 26428377 10.1007/978-3-319-22834-1_8

[11] Murata T. Regulation of Epstein-Barr virus reactivation from latency. Microbiol Immunol. 2014;58(6):307–17. doi: 10.1111/1348-0421.12155 . 24786491 10.1111/1348-0421.12155

[12] Kenney SC, Mertz JE. Regulation of the latent-lytic switch in Epstein-Barr virus. Semin Cancer Biol. 2014;26:60–8. doi: 10.1016/j.semcancer.2014.01.002 ; 24457012 10.1016/j.semcancer.2014.01.002 PMC4048781

[13] Miller G, El-Guindy A, Countryman J, Ye J, Gradoville L. Lytic cycle switches of oncogenic human gammaherpesviruses. Adv Cancer Res. 2007;97:81–109. doi: 10.1016/S0065-230X(06)97004-3 . 17419942 10.1016/S0065-230X(06)97004-3

[14] Murata T, Tsurumi T. Switching of EBV cycles between latent and lytic states. Rev Med Virol. 2014;24(3):142–53. doi: 10.1002/rmv.1780 . 24339346 10.1002/rmv.1780

[15] Hagemeier SR, Barlow EA, Meng Q, Kenney SC. The cellular ataxia telangiectasia-mutated kinase promotes epstein-barr virus lytic reactivation in response to multiple different types of lytic reactivation-inducing stimuli. Journal of virology. 2012;86(24):13360–70. doi: 10.1128/JVI.01850-12 ; 23015717 10.1128/JVI.01850-12 PMC3503132

[16] Chua HH, Chiu HY, Lin SJ, Weng PL, Lin JH, Wu SW, et al p53 and Sp1 cooperate to regulate the expression of Epstein-Barr viral Zta protein. Journal of medical virology. 2012;84(8):1279–88. doi: 10.1002/jmv.23316 . 22711357 10.1002/jmv.23316

[17] Hagemeier SR, Dickerson SJ, Meng Q, Yu X, Mertz JE, Kenney SC. Sumoylation of the Epstein-Barr virus BZLF1 protein inhibits its transcriptional activity and is regulated by the virus-encoded protein kinase. Journal of virology. 2010;84(9):4383–94. doi: 10.1128/JVI.02369-09 ; 20181712 10.1128/JVI.02369-09 PMC2863741

[18] Adamson AL, Kenney S. Epstein-barr virus immediate-early protein BZLF1 is SUMO-1 modified and disrupts promyelocytic leukemia bodies. Journal of virology. 2001;75(5):2388–99. doi: 10.1128/JVI.75.5.2388-2399.2001 ; 11160742 10.1128/JVI.75.5.2388-2399.2001 PMC114822

[19] Deng Z, Chen CJ, Zerby D, Delecluse HJ, Lieberman PM. Identification of acidic and aromatic residues in the Zta activation domain essential for Epstein-Barr virus reactivation. J Virol. 2001;75(21):10334–47. doi: 10.1128/JVI.75.21.10334-10347.2001 ; 11581402 10.1128/JVI.75.21.10334-10347.2001 PMC114608

[20] Sato Y, Kamura T, Shirata N, Murata T, Kudoh A, Iwahori S, et al Degradation of phosphorylated p53 by viral protein-ECS E3 ligase complex. PLoS pathogens. 2009;5(7):e1000530 doi: 10.1371/journal.ppat.1000530 ; 19649319 10.1371/journal.ppat.1000530 PMC2712087

[21] Zhang Q, Gutsch D, Kenney S. Functional and physical interaction between p53 and BZLF1: implications for Epstein-Barr virus latency. Molecular and cellular biology. 1994;14(3):1929–38. ; 8114724 10.1128/mcb.14.3.1929-1938.1994 PMC358551

[22] Sompallae R, Gastaldello S, Hildebrand S, Zinin N, Hassink G, Lindsten K, et al Epstein-barr virus encodes three bona fide ubiquitin-specific proteases. Journal of virology. 2008;82(21):10477–86. doi: 10.1128/JVI.01113-08 ; 18715931 10.1128/JVI.01113-08 PMC2573217

[23] Gastaldello S, Chen X, Callegari S, Masucci MG. Caspase-1 promotes Epstein-Barr virus replication by targeting the large tegument protein deneddylase to the nucleus of productively infected cells. PLoS Pathog. 2013;9(10):e1003664 doi: 10.1371/journal.ppat.1003664 ; 24130483 10.1371/journal.ppat.1003664 PMC3795028

[24] Gastaldello S, Hildebrand S, Faridani O, Callegari S, Palmkvist M, Di Guglielmo C, et al A deneddylase encoded by Epstein-Barr virus promotes viral DNA replication by regulating the activity of cullin-RING ligases. Nature cell biology. 2010;12(4):351–61. doi: 10.1038/ncb2035 . 20190741 10.1038/ncb2035

[25] Flick K, Kaiser P. Set them free: F-box protein exchange by Cand1. Cell research. 2013;23(7):870–1. doi: 10.1038/cr.2013.55 ; 23609796 10.1038/cr.2013.55 PMC3698631

[26] Liu J, Furukawa M, Matsumoto T, Xiong Y. NEDD8 modification of CUL1 dissociates p120(CAND1), an inhibitor of CUL1-SKP1 binding and SCF ligases. Molecular cell. 2002;10(6):1511–8. . 12504025 10.1016/s1097-2765(02)00783-9

[27] Zheng J, Yang X, Harrell JM, Ryzhikov S, Shim EH, Lykke-Andersen K, et al CAND1 binds to unneddylated CUL1 and regulates the formation of SCF ubiquitin E3 ligase complex. Molecular cell. 2002;10(6):1519–26. . 12504026 10.1016/s1097-2765(02)00784-0

[28] Tikhmyanova N, Schultz DC, Lee T, Salvino JM, Lieberman PM. Identification of a new class of small molecules that efficiently reactivate latent Epstein-Barr Virus. ACS chemical biology. 2014;9(3):785–95. doi: 10.1021/cb4006326 ; 24028149 10.1021/cb4006326 PMC4159771

[29] Root DE, Hacohen N, Hahn WC, Lander ES, Sabatini DM. Genome-scale loss-of-function screening with a lentiviral RNAi library. Nat Methods. 2006;3(9):715–9. doi: 10.1038/nmeth924 . 16929317 10.1038/nmeth924

[30] Yu X, Wang Z, Mertz JE. ZEB1 regulates the latent-lytic switch in infection by Epstein-Barr virus. PLoS Pathog. 2007;3(12):e194 doi: 10.1371/journal.ppat.0030194 ; 18085824 10.1371/journal.ppat.0030194 PMC2134958

[31] Kraus RJ, Perrigoue JG, Mertz JE. ZEB negatively regulates the lytic-switch BZLF1 gene promoter of Epstein-Barr virus. J Virol. 2003;77(1):199–207. doi: 10.1128/JVI.77.1.199-207.2003 ; 12477825 10.1128/JVI.77.1.199-207.2003 PMC140584

[32] Mao X, Gluck N, Chen B, Starokadomskyy P, Li H, Maine GN, et al COMMD1 (copper metabolism MURR1 domain-containing protein 1) regulates Cullin RING ligases by preventing CAND1 (Cullin-associated Nedd8-dissociated protein 1) binding. J Biol Chem. 2011;286(37):32355–65. ; 21778237 10.1074/jbc.M111.278408 PMC3173175

[33] Kanakry JA, Ambinder RF. EBV-related lymphomas: new approaches to treatment. Current treatment options in oncology. 2013;14(2):224–36. doi: 10.1007/s11864-013-0231-y ; 23549980 10.1007/s11864-013-0231-y PMC3670765

[34] Tanaka T, Nakatani T, Kamitani T. Inhibition of NEDD8-conjugation pathway by novel molecules: potential approaches to anticancer therapy. Mol Oncol. 2012;6(3):267–75. doi: 10.1016/j.molonc.2012.01.003 ; 22306028 10.1016/j.molonc.2012.01.003 PMC3826113

[35] Duda DM, Scott DC, Calabrese MF, Zimmerman ES, Zheng N, Schulman BA. Structural regulation of cullin-RING ubiquitin ligase complexes. Current opinion in structural biology. 2011;21(2):257–64. doi: 10.1016/j.sbi.2011.01.003 ; 21288713 10.1016/j.sbi.2011.01.003 PMC3151539

[36] Pierce NW, Lee JE, Liu X, Sweredoski MJ, Graham RL, Larimore EA, et al Cand1 promotes assembly of new SCF complexes through dynamic exchange of F box proteins. Cell. 2013;153(1):206–15. doi: 10.1016/j.cell.2013.02.024 ; 23453757 10.1016/j.cell.2013.02.024 PMC3656483

[37] Shibata E, Abbas T, Huang X, Wohlschlegel JA, Dutta A. Selective ubiquitylation of p21 and Cdt1 by UBCH8 and UBE2G ubiquitin-conjugating enzymes via the CRL4Cdt2 ubiquitin ligase complex. Molecular and cellular biology. 2011;31(15):3136–45. doi: 10.1128/MCB.05496-11 ; 21628527 10.1128/MCB.05496-11 PMC3147600

[38] Hayashi A, Suenaga N, Shiomi Y, Nishitani H. PCNA-dependent ubiquitination of Cdt1 and p21 in mammalian cells. Methods Mol Biol. 2014;1170:367–82. doi: 10.1007/978-1-4939-0888-2_19 . 24906324 10.1007/978-1-4939-0888-2_19

[39] Guardavaccaro D, Pagano M. Oncogenic aberrations of cullin-dependent ubiquitin ligases. Oncogene. 2004;23(11):2037–49. doi: 10.1038/sj.onc.1207413 . 15021891 10.1038/sj.onc.1207413

[40] Abbas T, Sivaprasad U, Terai K, Amador V, Pagano M, Dutta A. PCNA-dependent regulation of p21 ubiquitylation and degradation via the CRL4Cdt2 ubiquitin ligase complex. Genes Dev. 2008;22(18):2496–506. doi: 10.1101/gad.1676108 ; 18794347 10.1101/gad.1676108 PMC2546691

[41] Geng F, Wenzel S, Tansey WP. Ubiquitin and proteasomes in transcription. Annual review of biochemistry. 2012;81:177–201. doi: 10.1146/annurev-biochem-052110-120012 ; 22404630 10.1146/annurev-biochem-052110-120012 PMC3637986

[42] Muratani M, Tansey WP. How the ubiquitin-proteasome system controls transcription. Nat Rev Mol Cell Biol. 2003;4(3):192–201. doi: 10.1038/nrm1049 . 12612638 10.1038/nrm1049

[43] Kayukawa K, Kitajima Y, Tamura T. TBP-interacting protein TIP120A is a new global transcription activator with bipartite functional domains. Genes to cells: devoted to molecular & cellular mechanisms. 2001;6(2):165–74. . 11260261 10.1046/j.1365-2443.2001.00407.x

[44] Shiraishi S, Zhou C, Aoki T, Sato N, Chiba T, Tanaka K, et al TBP-interacting protein 120B (TIP120B)/cullin-associated and neddylation-dissociated 2 (CAND2) inhibits SCF-dependent ubiquitination of myogenin and accelerates myogenic differentiation. The Journal of biological chemistry. 2007;282(12):9017–28. 17242400 10.1074/jbc.M611513200

[45] Qian TM, Zhao LL, Wang J, Li P, Qin J, Liu YS, et al miR-148b-3p promotes migration of Schwann cells by targeting cullin-associated and neddylation-dissociated 1. Neural Regen Res. 2016;11(6):1001–5. doi: 10.4103/1673-5374.184504 ; 27482232 10.4103/1673-5374.184504 PMC4962562

[46] Riley KJ, Rabinowitz GS, Yario TA, Luna JM, Darnell RB, Steitz JA. EBV and human microRNAs co-target oncogenic and apoptotic viral and human genes during latency. EMBO J. 2012;31(9):2207–21. doi: 10.1038/emboj.2012.63 ; 22473208 10.1038/emboj.2012.63 PMC3343464

[47] Mandage R, Telford M, Rodriguez JA, Farre X, Layouni H, Marigorta UM, et al Genetic factors affecting EBV copy number in lymphoblastoid cell lines derived from the 1000 Genome Project samples. PLoS One. 2017;12(6):e0179446 doi: 10.1371/journal.pone.0179446 . 28654678 10.1371/journal.pone.0179446 PMC5487016

[48] Oladghaffari M, Islamian JP, Baradaran B, Monfared AS. MLN4924 therapy as a novel approach in cancer treatment modalities. J Chemother. 2016;28(2):74–82. doi: 10.1179/1973947815Y.0000000066 . 26292710 10.1179/1973947815Y.0000000066

[49] Chen HS, Wikramasinghe P, Showe L, Lieberman PM. Cohesins repress Kaposi's sarcoma-associated herpesvirus immediate early gene transcription during latency. J Virol. 2012;86(17):9454–64. doi: 10.1128/JVI.00787-12 ; 22740398 10.1128/JVI.00787-12 PMC3416178

[50] Storey JD, Tibshirani R. Statistical significance for genomewide studies. Proc Natl Acad Sci U S A. 2003;100(16):9440–5. doi: 10.1073/pnas.1530509100 ; 12883005 10.1073/pnas.1530509100 PMC170937

[51] Huang da W, Sherman BT, Lempicki RA. Systematic and integrative analysis of large gene lists using DAVID bioinformatics resources. Nat Protoc. 2009;4(1):44–57. doi: 10.1038/nprot.2008.211 . 19131956 10.1038/nprot.2008.211

