# Supplementary figures and images for: Small molecule perturbation of the CAND1-Cullin1-ubiquitin cycle stabilizes p53 and triggers Epstein-Barr virus reactivation
Source: PLoS Pathog. 2017 Jul 17;13(7):e1006517. doi: 10.1371/journal.ppat.1006517 (PMC5531659; doi:10.1371/journal.ppat.1006517)

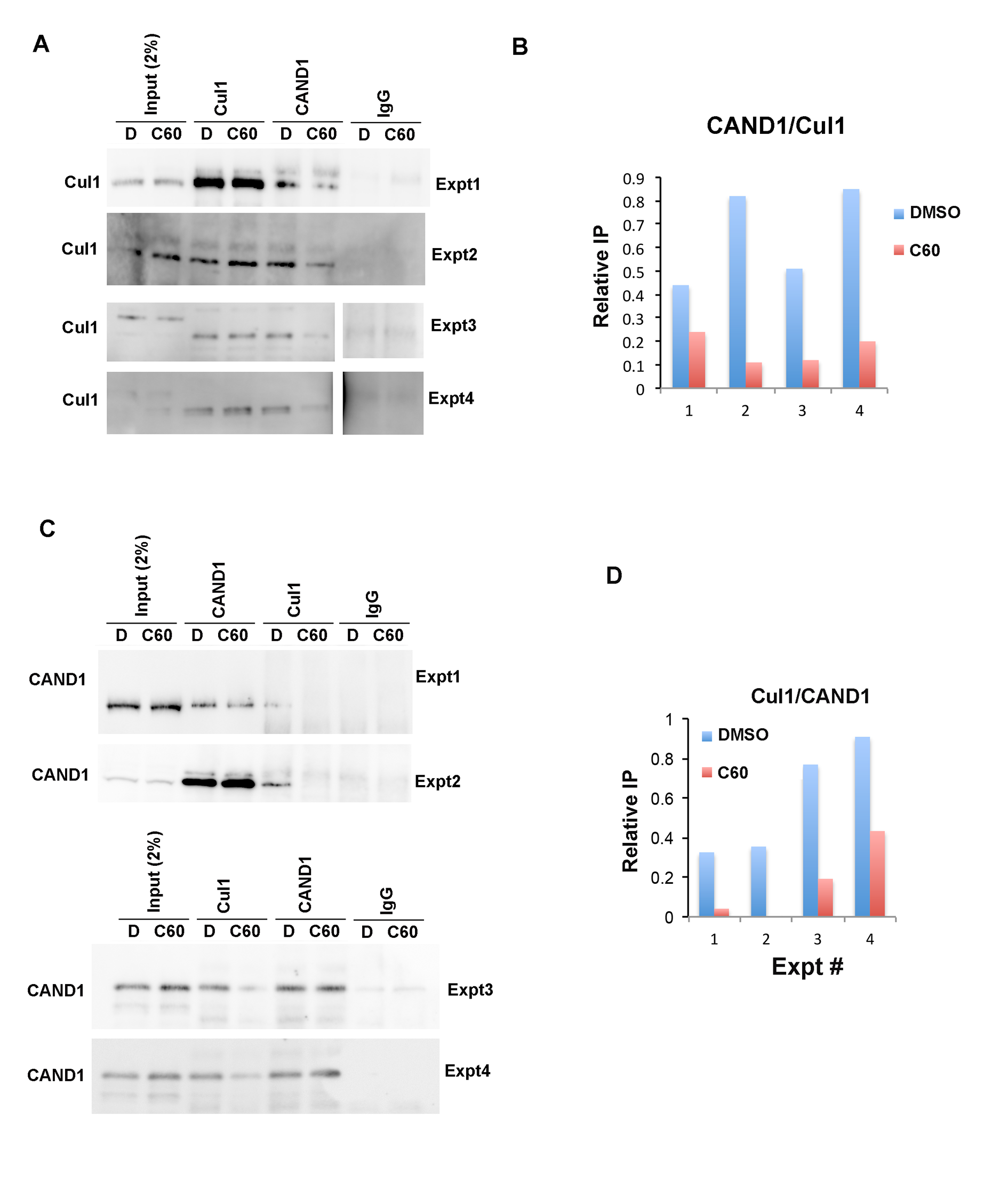

Supplement: S1 Fig — Total cell lysates from Mutu I cells treated with DMSO or 1 μM C60 were subject to IP with antibody to Cul 1, CAND1, or control IgG, and then assayed by Western blot for Cul 1 (panel A), or CAND1 (panel C). Blots for 4 independent biological replicates (Expt 1–4) are shown. Quantitative densitometry is shown for each blot as intensity of Cul 1 in IP CAND1 relative to IP Cul 1 for (panel B) or for CAND1 in IP Cul 1 relative to IP CAND1 (panel D) each experiment 1–4. (TIF) [file ppat.1006517.s001.tif]

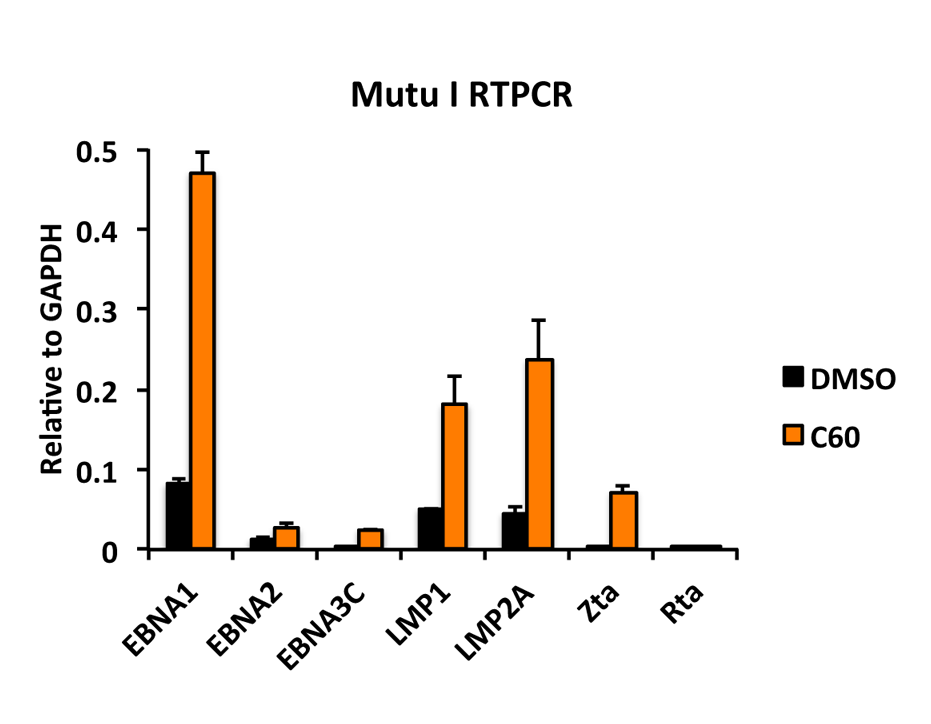

Supplement: S2 Fig — RT-qPCR analysis for EBV gene transcription (as indicated) in Mutu I cells treated with either DMSO (black), or 5 μM C60 (orange) for 48 hours. (TIF) [file ppat.1006517.s002.tif]

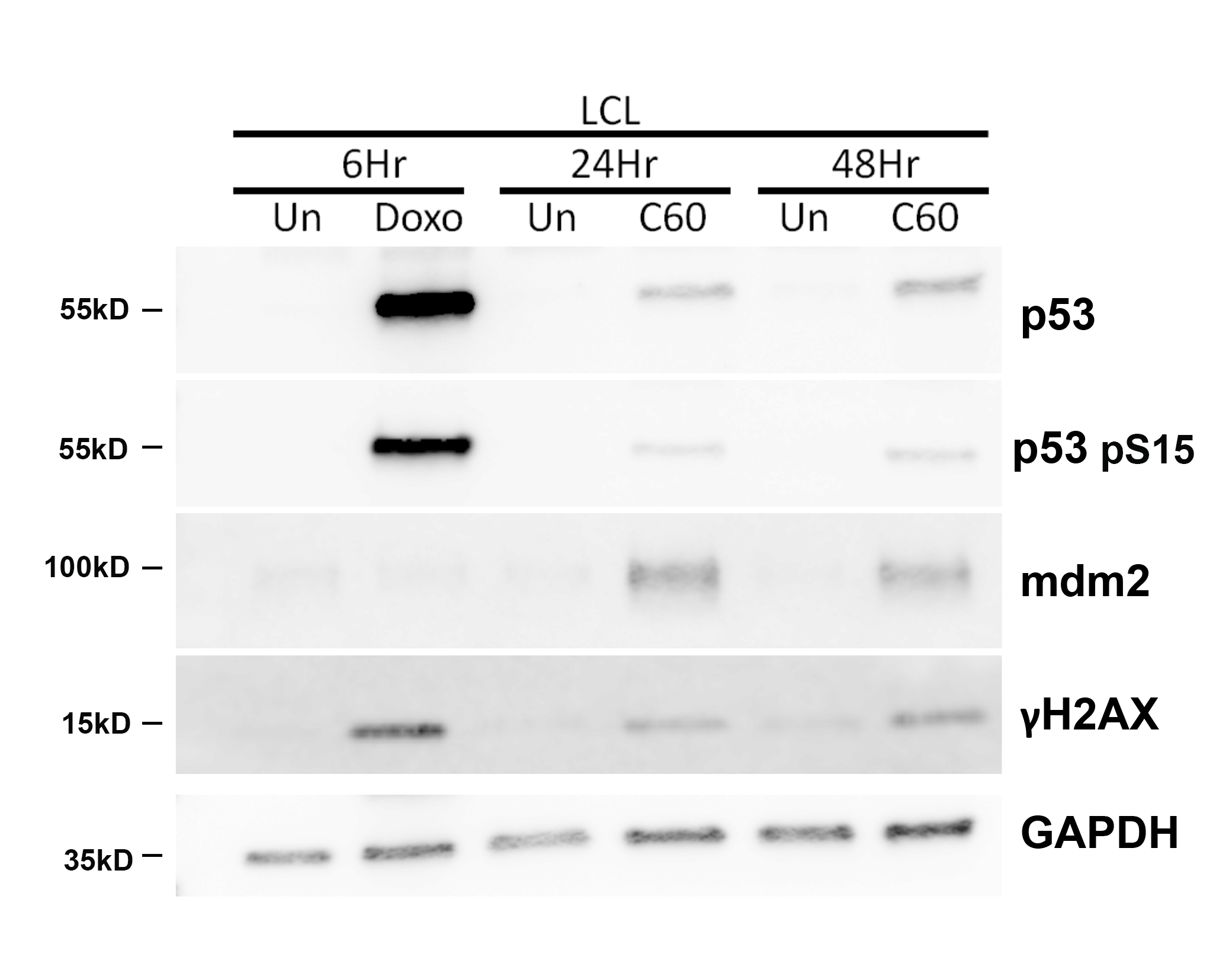

Supplement: S3 Fig — LCLs were treated with 2 μM doxorubicin for 6 hrs, or 5 μM C60 for 24 of 48 hrs and assayed by Western blot for total p53, p53 pS15, γH2AX, or GAPDH. (TIF) [file ppat.1006517.s003.tif]
